# Electron transport in acetate-grown *Methanosarcina acetivorans*

**DOI:** 10.1186/1471-2180-11-165

**Published:** 2011-07-24

**Authors:** Mingyu Wang, Jean-Francois Tomb, James G Ferry

**Affiliations:** 1 Department of Biochemistry and Molecular Biology, Eberly College of Science, The Pennsylvania State University, University Park, Pennsylvania 16802-4500, USA; 2 E. I. DuPont de Nemours Company, Central Research and Development, Experimental Station, Wilmington, Delaware 19880, USA

## Abstract

**Background:**

Acetate is the major source of methane in nature. The majority of investigations have focused on acetotrophic methanogens for which energy-conserving electron transport is dependent on the production and consumption of H_2 _as an intermediate, although the great majority of acetotrophs are unable to metabolize H_2_. The presence of cytochrome *c *and a complex (Ma-Rnf) homologous to the Rnf (***R***hodobacter ***n***itrogen ***f***ixation) complexes distributed in the domain *Bacteria *distinguishes non-H_2_-utilizing *Methanosarcina acetivorans *from H_2_-utilizing species suggesting fundamentally different electron transport pathways. Thus, the membrane-bound electron transport chain of acetate-grown *M. acetivorans *was investigated to advance a more complete understanding of acetotrophic methanogens.

**Results:**

A component of the CO dehydrogenase/acetyl-CoA synthase (CdhAE) was partially purified and shown to reduce a ferredoxin purified using an assay coupling reduction of the ferredoxin to oxidation of CdhAE. Mass spectrometry analysis of the ferredoxin identified the encoding gene among annotations for nine ferredoxins encoded in the genome. Reduction of purified membranes from acetate-grown cells with ferredoxin lead to reduction of membrane-associated multi-heme cytochrome *c *that was re-oxidized by the addition of either the heterodisulfide of coenzyme M and coenzyme B (CoM-S-S-CoB) or 2-hydoxyphenazine, the soluble analog of methanophenazine (MP). Reduced 2-hydoxyphenazine was re-oxidized by membranes that was dependent on addition of CoM-S-S-CoB. A genomic analysis of *Methanosarcina thermophila*, a non-H_2_-utilizing acetotrophic methanogen, identified genes homologous to cytochrome *c *and the Ma-Rnf complex of *M. acetivorans*.

**Conclusions:**

The results support roles for ferredoxin, cytochrome *c *and MP in the energy-conserving electron transport pathway of non-H_2_-utilizing acetotrophic methanogens. This is the first report of involvement of a cytochrome *c *in acetotrophic methanogenesis. The results suggest that diverse acetotrophic *Methanosarcina *species have evolved diverse membrane-bound electron transport pathways leading from ferredoxin and culminating with MP donating electrons to the heterodisulfide reductase (HdrDE) for reduction of CoM-S-S-CoB.

## Background

The decomposition of complex organic matter to methane (biomethanation) in diverse anaerobic habitats of Earth's biosphere involves an anaerobic microbial food chain comprised of distinct metabolic groups, the first of which metabolizes the complex organic matter primarily to acetate and also formate or H_2 _that are growth substrates for two distinct methane-producing groups (methanogens) [1]. The methyl group of acetate contributes most of the methane produced in the biomethanation process *via *the aceticlastic pathway whereas the remainder originates primarily from the reduction of CO_2 _with electrons derived from the oxidation of formate or H_2 _in the CO_2_-reduction pathway [2,3]. Smaller, albeit significant, amounts of methane derive from the methyl groups of methanol, methylamines and dimethylsulfide [1].

Only two genera of aceticlastic methanogens have been described, *Methanosarcina *and *Methanosaeta *[2]. In both genera, the CO dehydrogenase/acetyl-CoA complex (Cdh) cleaves activated acetate into methyl and carbonyl groups. The methyl group is transferred to coenzyme M (HS-CoM) producing CH_3_-S-CoM that is reductively demethylated to methane with electrons donated by coenzyme B (HS-CoB). The heterodisulfide CoM-S-S-CoB is a product of the demethylation reaction that is reduced to the sulfhydryl forms of the cofactors by heterodisulfide reductase (Hdr). The proton gradient driving ATP synthesis is generated *via *a membrane-bound electron transport chain originating with oxidation of the carbonyl group of acetate by Cdh and terminating with reduction of CoM-S-S-CoB by Hdr. Although the pathway of carbon flow from the methyl group of acetate to methane is understood for both aceticlastic genera, the understanding of electron transport coupled to generation of the proton gradient is incomplete. The majority of investigations have focused on *Methanosarcina barkeri *and *Methanosarcina mazei *for which electron transport is dependent on the production and consumption of H_2 _as an intermediate, although the great majority of *Methanosarcina *species [4] and all *Methanosaeta *species are unable to metabolize H_2_.

In the H_2_-metabolizing *Methanosarcina *species investigated, a ferredoxin accepts electrons from Cdh [5,6] and donates to a membrane-bound Ech hydrogenase complex that produces H_2 _and generates a proton gradient for ATP synthesis [7-9]. A hypothesis has been advanced wherein H_2 _is re-oxidized by another membrane-bound hydrogenase (Vho) that transfers electrons to methanophenazine (MP), a quinone-like electron carrier [9]. In the model, MP donates electrons to the heterodisulfide reductase HdrDE accompanied by translocation of protons which further contributes to ATP synthesis.

An electron transport chain has been hypothesized for the marine isolate *Methanosarcina acetivorans*, the only non-H_2_-metabolizing acetotrophic methanogen for which the genome is sequenced. Although encoding Cdh, the genome does not encode Ech hydrogenase [10,11]. Furthermore, in contrast to all H_2_-utilizing aceticlastic *Methanosarcina *species investigated [12], acetate-grown *M. acetivorans *synthesizes a six-subunit complex (Ma-Rnf) [13] encoded within a co-transcribed eight-gene (MA0658-0665) cluster with high identity to membrane-bound Rnf (**R***hodobacter ****n***itrogen ***f***ixation) complexes from the domain *Bacteria*. It is hypothesized that the Ma-Rnf complex plays an essential role in the electron transport chain, generating a sodium gradient that is exchanged for a proton gradient driving ATP synthesis [13]. Consistent with this idea, it was recently shown that the six-subunit Rnf complex from *Acetobacterium woodii *of the domain *Bacteria *couples electron transport from reduced ferredoxin to NAD^+ ^with the generation of a sodium gradient [14]. Remarkably, the Ma-Rnf complex of *M. acetivorans *is co-transcribed with a gene (MA0658) encoding a multi-heme cytochrome *c*, and another flanking gene (MA0665) encoding a hypothetical membrane integral protein with unknown function [13]. Indeed, the cytochrome *c *was shown to be synthesized in high levels of acetate-grown cells where it completely dominates the UV-visible spectrum of the purified membranes and is distinguishable from *b*-type cytochromes [13]. Furthermore, it was recently reported (A. M. Guss and W. W. Metcalf, unpublished results) that a six-subunit Ma-Rnf/cytochrome *c *(Δ*MA0658-0665*) deletion mutant of *M. acetivorans *fails to grow with acetate [15]. However, biochemical evidence necessary to support the hypothesized role of cytochrome *c *has not been forthcoming. The only other report of cytochromes *c *in methanogens is for the H_2_-metabolizing species *Methanosarcina mazei *(*f. Methanosarcina *strain Gö1) grown with methanol [16].

The freshwater isolate *Methanosarcina thermophila *is the only non-H_2_-metabolizing acetotrophic methanogen for which electron transport components have been investigated biochemically [17]. Like H_2_-metabolizing *Methanosarcina *species, ferredoxin mediates electron transfer between Cdh and the membrane-bound electron transport chain in which a cytochrome *b *participates and dominates the UV-visible absorbance spectrum of membranes. It is also reported that MP is the electron donor to HdrDE [18]. Electron carriers other than cytochrome *b *that participate between ferredoxin and MP were not identified. Importantly, no evidence for participation of Ma-Rnf or cytochrome *c *was reported. Homologs encoding an Ma-Rnf complex and cytochrome *c *are absent in the sequenced genome of *Methanosaeta thermophila *suggesting yet another novel electron transport chain that functions in the conversion of acetate to methane in this non-H_2_-metabolizing genus [19]. Clearly, diverse electron transport pathways have evolved in diverse acetotrophic methanogens necessitating biochemical investigations of representative species.

The absence of Ech hydrogenase and the demonstrated presence of the Ma-Rnf complex and cytochrome *c *that is elevated in acetate- *versus *methanol- grown cells [13] suggests that electron transport of the non-H_2_-metabolizing marine isolate *M. acetivorans *is decidedly dissimilar from the genus *Methanosaeta *and H_2_-metabolizing acetotrophic species of the genus *Methanosarcina*. However, a biochemical investigation essential to support the role of electron carriers has not been reported for *M. acetivorans*. Here we report evidence indicating roles for ferredoxin, cytochrome *c *and MP in electron transport of acetate-grown *M. acetivorans*. The results underscore the diversity of electron transport pathways in acetotrophic methanogens and contribute to a more complete understanding of acetotrophic methanogenesis.

## Results

### The electron acceptor for the CO dehydrogenase/acetyl-CoA complex of *M. acetivorans*

The Cdh from acetate-grown *M. acetivorans *was purified to ascertain the electron acceptor that initiates electron transport. The Cdh complex purified from the H_2_-metabolizing acetotrophic species *Methanosarcina barkeri *contains five-subunits (CdhABCDE) [20] of which the CdhAE component oxidizes CO derived from the carbonyl group of acetate [21]. The genome of *M. acetivorans *is annotated with duplicate Cdh gene clusters [10], each encoding five subunits homologous to the Cdh subunits of *M. barkeri*. Previous proteomic analyses of acetate-grown *M. acetivorans *identified subunits CdhA, CdhB and CdhC from one cluster (MA1011-16) and CdhA, CdhB CdhC and CdhE from the other (MA3860-65) [22]. The purification was monitored by following the CO-dependent reduction of methyl viologen. SDS PAGE of the purified enzyme showed bands with molecular masses of 16 kDa and 85 kDa consistent with the predicted values for the CdhA and CdhE subunits encoded in the genome. Mass spectrometry of the protein bands identified the CdhA and CdhE subunits encoded by both Cdh gene clusters consistent with previous proteomic analyses that indicated up-regulation of both clusters in acetate- *versus *methanol-grown cells [22].

**Figure 1:**
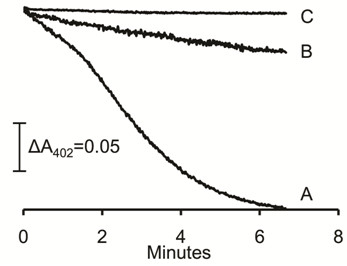
**Reduction of ferredoxin by CdhAE**. The 70-μl reaction mixture consisted of 2.2 μg of CdhAE and 28 μM (final concentration) of ferredoxin contained in 50 mM MOPS buffer (pH 6.8) under 1 atm CO. The reaction was initiated with CdhAE. A, complete reaction mixture initial absorbance 0.61. B, reaction mixture minus CdhAE, initial absorbance 0.72. C, reaction mixture minus ferredoxin, initial absorbance 0.72. The reduction of ferredoxin was followed by the decrease in absorbance at 402 nm.

Ferredoxin from acetate-grown cells of *M. acetivorans *was purified as described in the Methods section to determine if it accepts electrons from the partially purified CdhAE components thereby initiating electron transport. Mass spectrometry analysis of the purified ferredoxin detected only one protein identified as the product of MA0431 previously annotated as a 2 × [4Fe-4S] ferredoxin [23]. The UV-visible absorption spectrum of the as-purified protein was typical of ferredoxins with an absorption maximum at 402 nm that decreased upon reduction with dithionite (Additional file 1, Figure S1). The genome of *M. acetivorans *is annotated with nine genes encoding ferredoxins, a phylogenetic analysis of which is shown in Additional file 2, Figure S2. The analysis revealed that the product of MA0431 is closely related to the 2 × [4Fe-4S] ferredoxin purified from acetate-grown cells of *M. thermophila *[24-27] and the ferredoxin up-regulated in acetate- *versus *methanol-grown *M. mazei *[28]. These three ferredoxins contain two CX_2_CX_2_CX_3_CP motifs typical of 2 × [4Fe-4S] ferredoxins and share high identity within a distinct clade (Additional file 2, Figure S2). Figure 1 shows CO-dependent reduction of the purified *M. acetivorans *ferredoxin catalyzed by the CdhAE components purified from *M. acetivorans*. These results suggest that ferredoxin isolated initiates the electron transport chain in both *M. acetivorans *and H_2_-metabolizing acetotrophic *Methanosarcina *species.

### Ferredoxin as the electron donor to the membrane-bound electron transport chain

**Figure 2:**
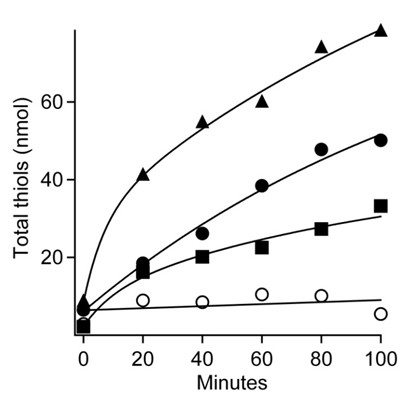
**Ferredoxin:heterodisulfide oxidoreductase activity of membranes**. The 100-μl reaction mixture consisted of 20 mM NADPH, 2 μg FNR (Sigma), the membrane fraction of acetate-grown cells (60 μg protein), 1.1 mM CoM-S-S-CoB and the indicated concentrations of ferredoxin contained in 50 mM MOPS buffer (pH 6.8). Total thiols were determined by the DTNB assay. Symbols: (filled triangles)1.2 μM ferredoxin, (filled circles) 0.6 μM ferredoxin, (filled squares) 0.3 μM ferredoxin, (open circles) minus ferredoxin.

The finding that ferredoxin is an electron acceptor for the CdhAE component of the Cdh complex of *M. acetivorans *raises the question whether it is the direct electron donor to membrane-bound electron carriers or if other soluble electron carriers are required to mediate electron transfer between ferredoxin and the membrane. This question was addressed in a system containing sucrose gradient-purified membranes and plant ferredoxin-NADPH reductase (FNR) to regenerate reduced ferredoxin that was purified from acetate-grown cells. The CO-dependent reduction of ferredoxin with CdhAE was not used to avoid binding of CO to high spin heme in cytochrome *c *and potentially inhibiting membrane-bound electron transport. The NADPH:CoM-S-S-CoB oxidoreductase activity was monitored by detecting the sulfhydryl groups of HS-CoM and HS-CoB (Figure 2). No significant activity was detected when each component of the reaction mixture was deleted individually including membranes. The dependence of the activity on purified membranes and the concentration of ferredoxin purified from acetate-grown *M. acetivorans *indicated a role for the ferredoxin in the direct transfer of electrons from CdhAE to the membrane-bound electron transport chain terminating with reduction of CoM-S-S-CoB by heterodisulfide reductase.

### Role of cytochrome *c *in the membrane-bound electron transport chain

**Figure 3:**
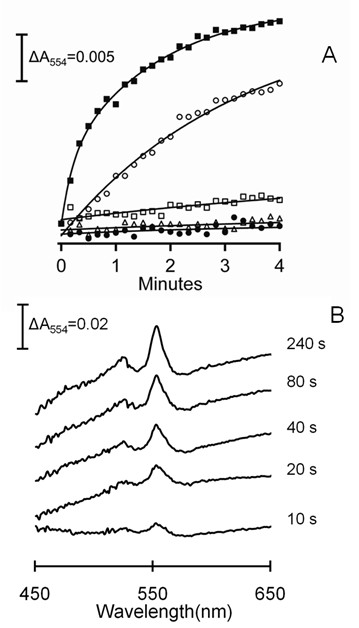
**Ferredoxin-dependent reduction of membrane-bound cytochrome *c***. The 100-μl reaction mixture consisted of purified membranes (300 μg protein), the indicated amount of ferredoxin, 1 μg FNR and 1 mM NADPH in 50 mM MOPS (pH 6.8). The reaction was initiated by addition of FNR (Sigma). The reduction of cytochrome *c *was followed at 554 nm. Panel A, time-course for the reduction of cytochrome *c*. Symbols: (filled squares) 4 μM ferredoxin; (open circles) 0.2 μM ferredoxin; (open squares) minus ferredoxin; (open triangles) minus FNR; (filled circles) minus NADPH. Panel B, reduced minus oxidized spectra recorded at the indicated times after initiation of the reaction containing 4 μM ferredoxin.

**Figure 4:**
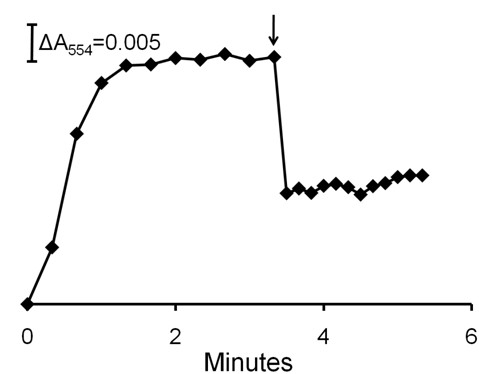
**Oxidation of membrane-bound cytochrome *c *by CoM-S-S-CoB**. The reduction of cytochrome *c *was performed as described in the caption to Figure 3. The 100-μl reaction mixture consisted of membranes (400 μg protein), 2 μM ferredoxin, 1 μg FNR and 1 mM NADPH. FNR was added at time zero and 0.32 mM (final concentration) CoM-S-S-CoB was added (arrow). Reduction and oxidation of cytochrome *c *was monitored by the absorbance at 554 nm.

It was previously documented [13] that purified membranes of acetate-grown *M. acetivorans *contain a multi-heme cytochrome *c *that clearly dominates the UV-visible spectrum of membranes from acetate-grown *M. acetivorans *with the major peak centered at 554 nm (Figure 3B). Absorbance at 554 nm increased on incubation of the membrane fraction with the reduced ferredoxin regenerating system indicating reduction of cytochrome *c *that was dependent on ferredoxin (Figure 3). Addition of CoM-S-S-CoB oxidized the reduced cytochrome (Figure 4) indicating that it is a component of the membrane-bound electron transport chain terminating with reduction of the heterodisulfide. The re-oxidation was too rapid to determine a rate and incomplete, albeit greater than 50%. The explanation for incomplete re-oxidation is unknown, although the result is nearly identical to that reported for the re-oxidation of cytochromes in the membrane fraction of methanol-grown *M. mazei *that was rapid and reached 40% re-oxidation [16]. This is the first report of cytochrome *c *involvement in the conversion of acetate to methane.

### Role of methanophenazine in the membrane-bound electron transport chain

**Figure 5:**
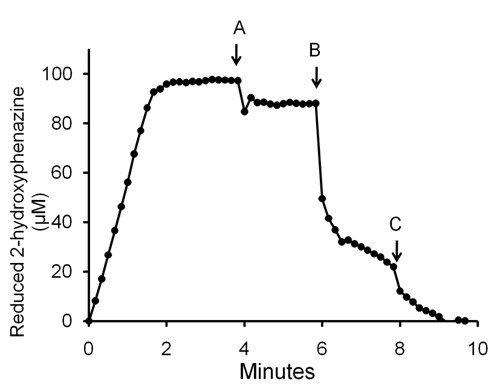
**Reduction of 2-hydroxyphenazine and re-oxidation dependent on membranes and CoM-S-S-CoB**. The 100-μl reaction mixture consisted of membranes (107 μg protein), 4 μM ferredoxin, 100 μM 2-hydroxyphenazine and CdhAE (40 μg) in 50 mM MOPS (pH 6.8) under 1 atm CO. The reduction and oxidation of 2-hydroxyphenazine was followed by the absorbance at 475 nm (ε_475 _= 2.5 mM^-1 ^cm^-1^). CdhAE was added to initiate the reduction at time zero. At point A the cuvette was flushed with 100% N_2 _and 2 μl of MOPS buffer (pH 6.8) was added. At points B and C, 2 μl of MOPS buffer (pH 6.8) containing CoM-S-S-CoB was added to the reaction reaching final concentrations of 240 and 480 μM.

The soluble analog of MP, 2-hydroxyphenazine, has been used to investigate the role of MP in methanogens [18,29]. The 2-hydroxyphenazine that was reduced in the presence of membranes with the CO/CdhAE ferredoxin regenerating system was re-oxidized upon addition of CoM-S-S-CoB (Figure 5) consistent with a membrane-bound heterodisulfide reductase. The re-oxidation generated a total of 239 μM free thiol groups in this representative experiment, a result that is in approximate agreement with the observed oxidation of 106 μM 2-hydroxyphenazine. Assuming a two-electron transfer from the MP analog, 212 μM free thiol groups would be expected. These results indicate that MP is a component of the membrane-bound electron transport chain terminating with reduction of CoM-S-S-CoB.

**Figure 6:**
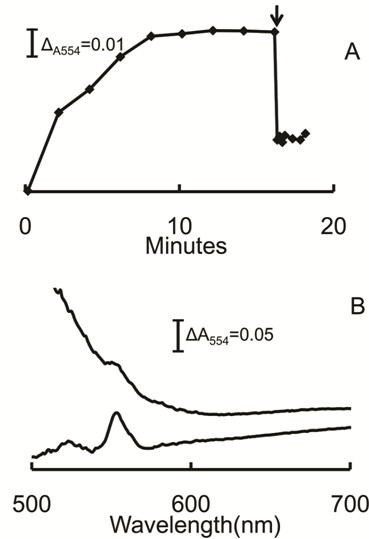
**Oxidation of membrane-bound cytochrome *c *by 2-hydroxyphenazine**. The 100-μl reaction mixture consisted of membranes (750 μg protein), 4 μM ferredoxin 1 mM NADPH and1 μg FNR contained in 50 mM MOPS buffer (pH 6.8). The reduction of cytochrome *c *was initiated by addition of FNR. The reduction and re-oxidation was monitored at 554 nm. When fully reduced, 200 μM 2-hydroxyphenazine (2 μl) was added (arrow). Panel A, time course for the reduction and re-oxidation by 2-hydroxyphenazine added at the arrow. Panel B, reduced minus oxidized UV-visible spectra of membranes before (lower trace) and after (upper trace) addition of 2-hydroxyphenazine.

The results implicating MP and cytochrome *c *in the membrane-bound electron transport chain presents the possibility of electron transfer between these carriers. The MP analog 2-hydroxyphenazine re-oxidized cytochrome *c *when added to membranes of acetate-grown cells previously reduced with ferredoxin (Figure 6). These results suggest that MP is either directly or indirectly linked to cytochrome *c*, a result further supporting the participation of MP and cytochrome *c *in the membrane-bound electron transport chain.

## Discussion

The overwhelming majority of methanogens capable of growth *via *conversion of the methyl group of acetate to methane do not metabolize H_2 _suggesting they employ an electron transport pathway distinct from that proposed for the few acetotrophic methanogens in which H_2 _is an obligatory intermediate. *M. acetivorans *is the ideal candidate to represent the majority of acetotrophic *Methanosarcina *species by virtue of its sequenced genome and published proteomic analyses leading to the hypothesis of a novel electron transport pathway for acetotrophic methanogens incapable of metabolizing H_2_. This, the first biochemical investigation of electron transport in *M. acetivorans*, has established roles for electron carriers that reveal both commonalities and differences in electron transport pathways of diverse acetotrophic *Methanosarcina *species.

**Figure 7:**
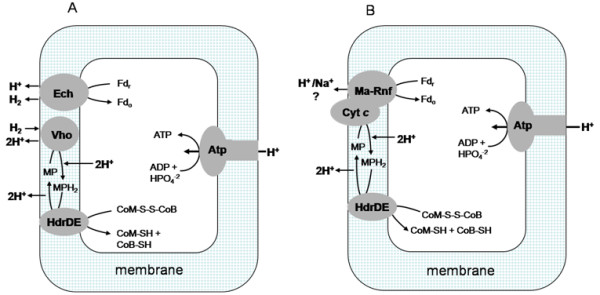
**Comparison of electron transport pathways for *Methanosarcina mazei *and *Methanosarcina barkeri versus Methanosarcina acetivorans***. Panel A, *M. mazei *and *M. barkeri*. Panel B, *M. acetivorans*. Ech, Ech hydrogenase; Fd_r_, ferredoxin reduced; Fd_o_, ferredoxin oxidized; Vho, Vho hydrogenase; MP, methanophenazine; HdrDE, heterodisulfide reductase; CoM-SH, coenzyme M; CoB-SH, coenzyme B; Atp, ATP synthase; Cyt *c*, cytochrome *c*; Ma-Rnf, Rnf complex from *M. acetivorans*; Mrp, putative sodium/proton antiporter.

Figure 7 compares the current understanding of electron transport for acetate-grown *M. acetivorans *with that for H_2_-metabolizing acetotrophic *Methanosarcina *species. In both pathways, the five-subunit CdhABCDE complex (not shown) cleaves the C-C and C-S bonds of acetyl-CoA releasing a methyl group and CO that is oxidized to CO_2 _with electrons transferred to ferredoxin. The CdhAE component of *M. acetivorans *was isolated independently from the other subunits and both copies encoded in the genome were represented. Although it was not possible to determine which CdhAE component reduced ferredoxin, the high percent identities (CdhA, MA1016 *vs*. MA3860 = 84% and CdhE, MA1015 *vs*. MA3861 = 82%) suggests it is the electron acceptor for either or both copies. In both pathways, ferredoxin is the electron donor to a membrane-bound electron transport chain that terminates with MP donating electrons to the heterodisulfide reductase HdrDE that catalyzes the reduction of CoB-S-S-CoM. Proteomic and genetic evidence [15,22] indicates that HdrDE functions in acetate-grown *M. acetivorans*. MP is the direct electron donor to HdrDE in acetate-grown cells of H_2_-metabolizing *Methanosarcina *species and the non-H_2_-metabolizing *M. thermophila *[18]. Thus, it is reasonable to postulate that MP is also the direct electron donor to HdrDE of *M. acetivorans*. However, the electron transport pathways of H_2_-metabolizing and non-H_2_-metabolizing species diverge significantly in electron transfer between ferredoxin and MP. In H_2_-metabolizing species, ferredoxin donates electrons to the membrane-bound Ech hydrogenase. A H_2 _cycling mechanism is postulated in which the H_2 _generated by Ech hydrogenase is re-oxidized by the MP-reducing Vho-type hydrogenase further contributing to the proton gradient [8]. Although the genome of *M. acetivorans *contains homologs of genes encoding Vho-type hydrogenases they are not expressed during growth with acetate [4], a result consistent with the absence of Ech hydrogenase and inability to metabolize H_2_. Instead, the results reported here support a role for cytochrome *c *mediating electron transport between ferredoxin and MP, although the identities of the direct electron donor and acceptor for cytochrome *c *remain unknown. The membrane location of cytochrome *c *is unknown; however, if on the outer aspect as for multi-heme cytochromes *c *in the domain *Bacteria*, ferredoxin would be an unlikely electron donor. The most probable electron donor to cytochrome *c *is the Ma-Rnf complex that is also hypothesized to accept electrons from ferredoxin in analogy to homologous Rnf complexes from the domain *Bacteria *[13,30]. In the absence of biochemical evidence, the proposed role for Ma-Rnf in electron transport is at least consistent with up-regulation in acetate *vs*. methanol-grown cells and the reported failure of an Ma-Rnf-cytochrome *c *deletion mutant (Δ*MA0658-0665*) of *M. acetivorans *to grow with acetate [15]. The proposed interaction of Ma-Rnf with cytochrome *c *is supported by co-transcription of the encoding genes and up-regulation in acetate- *vs*. methanol-grown cells [13]. A role for cytochrome *c *in the electron transport chain is also supported by results showing re-oxidation of cytochrome *c *upon addition of the MP analog 2-hydroxyphenazine to ferredoxin-reduced membranes, although an unknown carrier mediating electron transfer between cytochrome *c *and MP cannot be ruled out.

It was recently shown that the Rnf complex from *A. woodii *translocates sodium ions coupled to electron transfer from ferredoxin to NAD^+ ^[14]. In view of the potential sodium ion pumping function of Ma-Rnf, it is interesting to note that a multi-subunit sodium/proton antiporter (Mrp) is up-regulated in acetate-grown *M. acetivorans *and that the encoding genes are absent in H_2_-metabolizing *Methanosarcina *species [13]. Thus, it is tempting to speculate that Ma-Rnf generates a sodium gradient (high outside) that is exchanged for a proton gradient by Mrp. The only other coupling site is the reduction and oxidation of MP generating a proton gradient as proposed for H_2_-metabolizing *Methanosarcina *species (Figure 7). The role of a proton gradient driving ATP synthesis is consistent with the presence of a proton translocating ATP synthase in acetate-grown cells [13] recently shown to be the primary ATP synthase [31].

The available evidence indicates that the non-H_2_-metabolizing freshwater isolate *M. thermophila *also utilizes ferredoxin as electron donor to a membrane-bound electron transport chain involving cytochrome *b *and culminating with MP donating electrons to HdrDE [17,18,32]; however, a role for cytochrome *c *is not evident and other electron carriers have not been reported. Thus, based on current evidence, it appears that all acetotrophic *Methanosarcina *species have in common ferredoxin as electron donor to a membrane-bound electron transport chain terminating with MP donating electrons to HdrDE, although differ widely in membrane components transferring electrons from ferredoxin to MP. The evidence for involvement of HdrDE in acetate-grown cells is convincing; however, genes (MA2868, MA4236 and MA4237) homologous to those encoding the soluble HdrABC heterodisulfide reductase of CO_2_-reducing methanogens were shown to be up regulated in acetate- *versus *methanol-grown *M. acetivorans *[33]. This result is consistent with the previously reported increased abundance of HdrA encoded by MA2868 in acetate- *versus *methanol-grown *M. acetivorans *[22] which opens the possibility that the electron transport chain may terminate with both the membrane HdrDE or a soluble HdrABC heterodisulfide reductase.

Of the nine putative 2 × [4Fe-4S] ferredoxins annotated for the genome of *M. acetivorans*, only the ferredoxin encoded by MA0431 was purified from acetate-grown cells. While it cannot be ruled out that other ferredoxins are synthesized in acetate-grown cells, the results suggest that the ferredoxin encoded by MA0431 is at least dominant in acetate-grown cells. Of the nine putative 2 × [4Fe-4S] ferredoxins, the one purified from *M. acetivorans *is most closely related to that isolated from acetate-grown *M. thermophila *[26], a result suggesting it is the preferred electron acceptor of CdhAE in acetate-grown *Methanosarcina *species.

Interestingly, genes encoding subunits of Ma-Rnf or Ech hydrogenase are absent in the genome of the acetate-utilizing isolate *Methanosaeta thermophila *[19] that is also incapable of metabolizing H_2 _suggesting still other alternative electron transport pathways coupled to generation of ion gradients driving ATP synthesis in acetate-utilizing methanogens. The physiological significance of these diverse electron transport pathways is yet to be determined; however, it has been suggested that avoiding H_2 _is advantageous to the marine isolate *M. acetivorans *since sulfate reducing species that dominate this environment outcompete methanogens for H_2 _potentially disrupting electron transport [13]. It is important to note here that although *M. acetivorans *is incapable of growth with H_2_/CO_2 _it synthesizes all of the enzymes necessary for reduction of CO_2 _to methane and is capable of robust growth *via *the CO_2_-reduction pathway albeit with electrons derived from the oxidation of CO [34-36].

### Comparative analysis of the *M. thermophila *genome

*M. thermophila *is an acetotrophic *Methanosarcina *species incapable of metabolizing H_2 _[37,38]. Analysis of the genomic sequence revealed a gene cluster identical in arrangement and homologous to genes encoding the six subunits of Ma-Rnf and multi-heme cytochrome *c *of *M. acetivorans *with deduced sequence identities ranging from 86 to 98% (Additional file 3, Figure S3A). Alignments of the deduced sequences showed strict conservation of heme-binding, flavin binding and iron-sulfur binding motifs suggesting conserved functions (Additional file 3, Figure S3B). Although not conclusive, these results are consistent with a role for the Ma-Rnf complex and multi-heme cytochrome *c *in the electron transport pathway of *M. thermophila *grown with acetate. Furthermore, the genome of *M. thermophila *contains a gene cluster (Additional file 4, Figure S4) homologous to genes encoding the seven subunits of the sodium/proton antiporter (Mrp) that is up-regulated in acetate- *versus *methanol-grown cells of *M. acetivorans *and absent in the sequenced genomes of acetotrophic *Methanosarcina *species capable of metabolizing H_2_/CO_2 _[22,39].

## Conclusions

Although the majority of *Methanosarcina *species are unable to metabolize H_2_, electron transport has only been investigated in the few species for which H_2 _is an obligatory intermediate. *M. acetivorans *is proposed to utilize a fundamentally different electron transport pathway based on bio-informatic, proteomic and genetic approaches. However, the proposal has not been tested biochemically. The results indicate roles for ferredoxin, cytochrome *c *and MP in support of the proposed electron transport pathway. Further, this is the first report for involvement of a cytochrome *c *in acetotrophic methanogens. The results suggest that diverse acetotrophic *Methanosarcina *species have evolved diverse membrane-bound electron transport pathways leading from ferredoxin and culminating with MP donating electrons to HdrDE for reduction of CoM-S-S-CoB.

## Methods

### Materials

CoM-S-S-CoB was a kind gift of Dr. Jan Keltjens. 2-hydroxyphenazine was custom synthesized by Sigma-Aldrich (St. Louis, MO). All other chemicals were purchased from Sigma-Aldrich or VWR International (West Chester, PA). All chromatography columns, resins and pre-packed columns were purchased from GE Healthcare (Waukesha, WI).

### Preparation of cell extract and membranes

*M. acetivorans *[40] was cultured with acetate as described previously [41] and the cell paste was frozen at -80°C. All solutions were O_2_-free and manipulations were performed anaerobically in an anaerobic chamber (Coy Manufacturing, Ann Arbor, MI) containing 95% N_2 _and 5% H_2_. Frozen cells were thawed, re-suspended (1 g wet weight/ml buffer) in 50 mM MOPS buffer (pH 6.8) containing 10% (v/v) ethylene glycol and passed twice through a French pressure cell at 6.9 × 10^3 ^kPa. The lysate was centrifuged at 7,200 × *g *for 15 min to pellet cell debris and unbroken cells. Membranes were purified from the cell extract using a discontinuous sucrose gradient comprised of 2 ml 70% sucrose, 4 ml 30% sucrose and 1.5 ml 20% sucrose contained in 50 mM MOPS buffer (pH 6.8). A 2 ml volume of cell extract was overlaid on the gradient and centrifuged at 200,000 × *g *for 2 h in a Beckman type 50 Ti rotor. The brown band containing membranes at the 30% and 70% sucrose interface was collected and stored at -80°C until use.

### Purification of the αε component (CdhAE) of the CO dehydrogenase/acetyl-CoA synthase complex

All purification steps and biochemical assays were performed anaerobically in the anaerobic chamber. Crude cell extract of acetate-grown *M. acetivorans *was centrifuged at 200,000 × *g *for 2 h to pellet the membrane fraction. The supernatant solution (200 mg of protein in 10-ml) containing the soluble fraction was loaded onto a Q-Sepharose FF column (50 ml bed volume) equilibrated with 50 mM MOPS (pH 6.8). The column was developed with 500 ml of a 0-1.0 M NaCl linear gradient. Each 10 ml fraction was assayed for CO dehydrogenase activity by monitoring the CO-dependent reduction of methyl viologen as previously described [42]. The pooled fractions from the peak with the highest specific activity were concentrated 10-fold with a Vivacell 70 protein concentrator equipped with a 10-kDa cut off membrane (Sartorius Group, Göttingen, Germany). A 1.0 M solution of (NH_4_)_2_SO_4 _contained in 50 mM MOPS (pH 6.8) was added to the concentrated protein solution to final concentration of 900 mM and loaded onto a Phenyl-Sepharose FF (low sub) column (20-ml bed volume) equilibrated with 50 mM MOPS (pH 6.8) containing 1.0 M (NH_4_)_2_SO_4_. The column was developed with 100 ml of a 1.0-0.0 M (NH_4_)_2_SO_4 _decreasing linear gradient. Fractions from the peak of CO dehydrogenase activity were pooled and concentrated followed by addition of a volume of 50 mM MOPS (pH 6.8) to lower the (NH_4_)_2_SO_4 _concentration to below 100 mM and then loaded on a HiTrap Q-Sepharose HP column (5 ml bed volume) equilibrated with 50 mM MOPS buffer (pH 6.8). The column was developed with 50 ml of a 0-1.0 M NaCl linear gradient. The peak containing CO dehydrogenase activity that eluted at approximately 0.3 M NaCl was collected and stored at -80°C until use.

### Purification of ferredoxin

All purification steps and biochemical assays were performed anaerobically in the anaerobic chamber. Ferredoxin was assayed by the ability to couple CO oxidation by CdhAE to the reduction of metronidazole followed by the decrease in *A*_320 _(ε_320 _= 9300 M^-1 ^cm^-1^) similar to that described previously [27]. One unit of activity was the amount that reduced 1 μmol of metronidazole/min. The reaction mixture (100 μl) contained 100 μM metronidazole and 1-3 μg CdhAE in 50 mM Tris buffer (pH 8.0) to which 1-10 μl of the column fraction was added. The reaction was contained in an anaerobic cuvette flushed with 100% CO.

The soluble fraction of cell extract from acetate-grown *M. acetivorans *was loaded onto a Q-sepharose FF column (20 ml bed volume) equilibrated with 50 mM MOPS (pH 6.8) containing 10% (v/v) ethylene glycol. The column was developed with 200 ml of a 0-1.0 M linear NaCl gradient. The fraction with the highest activity was then diluted 10-fold with 50 mM MOPS (pH 6.8) containing 10% (v/v) ethylene glycol. The solution was loaded on a Mono Q column (1.7 ml bed volume) to which 10 ml of a 0-1.0 M NaCl linear gradient was applied. The fraction containing ferredoxin that eluted at 600 mM NaCl was loaded on a Sephadex G-75 gel filtration column (100 ml bed volume) and developed with 50 mM MOPS (pH 6.8) containing 10% (v/v) ethylene glycol and 150 mM NaCl. The peak containing the purified ferredoxin was concentrated to A_402 _> 0.2 with a Vivacell 70 protein concentrator equipped with a 5-kDa cutoff membrane and stored at -80°C until use. The protein concentration was estimated by the ratio of absorbance at 230 and 260 nm as described [43].

### Analytical

All protein concentrations except for ferredoxin were determined by the bicinchoninic acid assay using the reagent from Thermo Scientific, Inc.. Detection of the free sulfhydryl groups of CoM-SH and CoB-SH was performed as previously described [17]. The buffer used in the assay was 25 mM sodium acetate containing 1 mM DTNB (5,5'-dithiobis-(2-nitrobenzoic acid)). All assays in this study were performed anaerobically with vacuum degassed solutions contained in sealed cuvettes with the indicated atmosphere and at room temperature.

### Nucleotide sequence accession number

The sequences of DNA encoding Rnf and Mrp of *M. thermophila *have been deposited in the GenBank database under accession number JN173061, JN173062, JN173063, JN173064, JN173065, JN173066, JN173067, JN173068, JN173069, JN173070, JN173071, JN173072, JN173073, JN173074, JN173075 .

## Competing interests

The authors declare that they have no competing interests.

## Authors' contributions

MW carried out the biochemical studies, participated in sequence analysis and drafted the manuscript. J-F T carried out the genomic sequencing and sequence alignments. JGF conceived of the study, participated in its design and coordination, and finalized the manuscript. All authors read and approved the final manuscript.

## Supplementary Material

Additional file 1 **Figure S1. UV-visible absorption spectra of purified ferredoxin**. As-purified (--), dithionite reduced (...). The protein concentration was 20 μM. Click here for file

Additional file 2 **Figure S2. Phylogenetic analysis and sequence alignment of ferredoxins**. The *M. mazei *and *M. acetivorans *sequences, labeled with the prefix MA, were derived from the CMR database [23]. The *M. thermophila *(M.t.) sequence is published [26]. The sequence of the 2 × [4Fe-4S] *Clostridium pasteurianum *is published [44] and the sequence of the 2Fe-2S *Spinacia oleracea *ferredoxin was obtained from the NCBI database (accession number O04683). Panel A, Phylogenetic analysis of ferredoxins. The tree was constructed by the neighbor-joining method with the MEGA4 program [45]. Bootstrap values are shown at the nodes. Bar, evolutionary distance of 0.2. Panel B, Sequence alignment of ferredoxins from *Methanosarcina *species. Motifs predicted to ligate two 4Fe-4S clusters are highlighted. The alignment was performed with ClustalX2 [46]. Click here for file

Additional file 3 **Figure S3. Comparison of *rnf *genes between *Methanosarcina thermophila *and *Methanosarcina acetivorans***. Panel A. Organization of *rnf *genes in *Methanosarcina thermophila versus Methanosarcina acetivorans*. Numbers next to the arrows indicate deduced sequence identity. Panel B. Alignment of the deduced sequences of *rnf *genes between *Methanosarcina thermophila *(Mt) and *Methanosarcina acetivorans *(Ma). Highlighted are: conserved heme binding sites (CXXCH and CXXXCH) in Cyt *c*, the flavin binding motif (SGAT) in RnfG, and cysteine motifs binding iron-sulfur clusters in RnfC and RnfB. Click here for file

Additional file 4 **Figure S4. Alignment of *mrp *gene clusters between *Methanosarcina thermophila *and *Methanosarcina acetivorans***. Numbers next to the arrows indicate deduced sequence identity. Click here for file
