# Supplementary figures and images for: Electron transport in acetate-grown Methanosarcina acetivorans
Source: BMC Microbiol. 2011 Jul 24;11:165. doi: 10.1186/1471-2180-11-165 (PMC3160891; doi:10.1186/1471-2180-11-165)

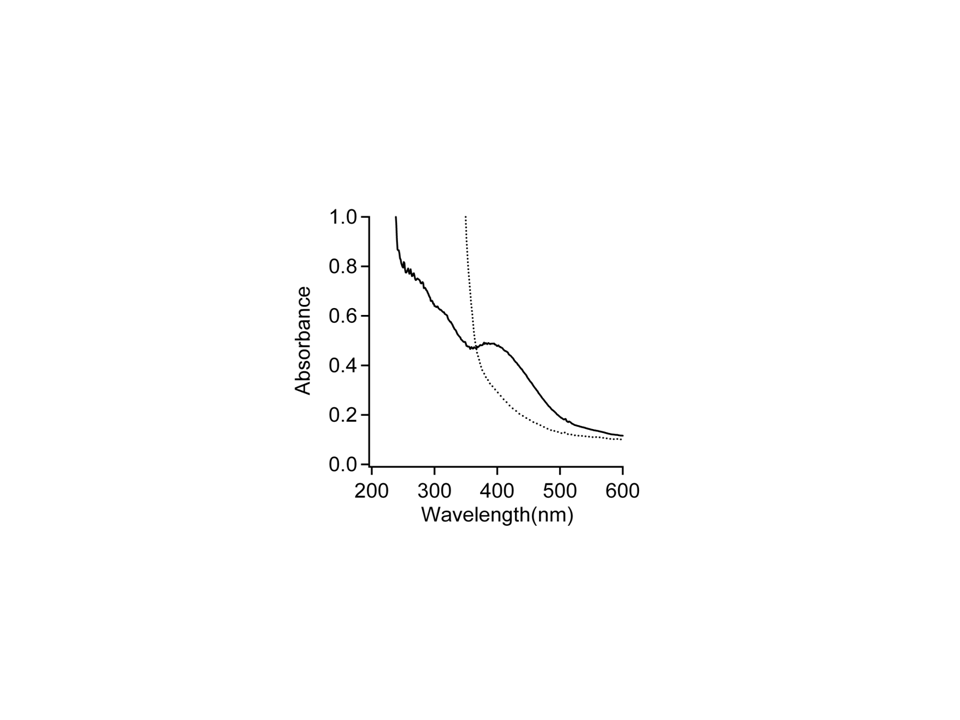

Supplement: Additional file 1 — Figure S1. UV-visible absorption spectra of purified ferredoxin. As-purified (--), dithionite reduced (...). The protein concentration was 20 μM. [file 1471-2180-11-165-S1.TIFF]

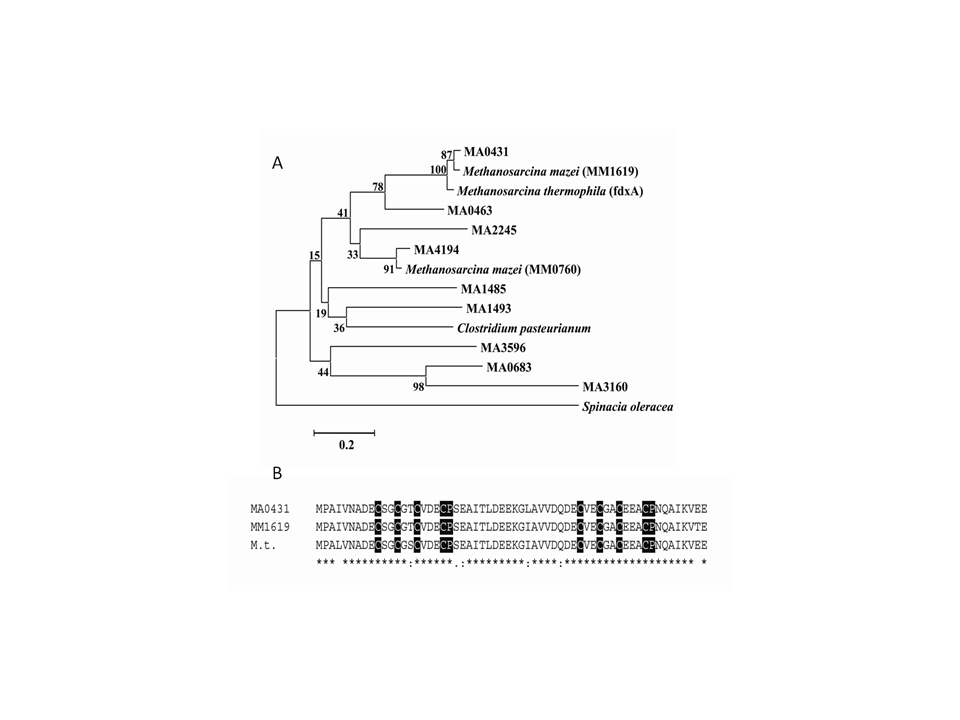

Supplement: Additional file 2 — Figure S2. Phylogenetic analysis and sequence alignment of ferredoxins. The M. mazei and M. acetivorans sequences, labeled with the prefix MA, were derived from the CMR database [23]. The M. thermophila (M.t.) sequence is published [26]. The sequence of the 2 × [4Fe-4S] Clostridium pasteurianum is published [44] and the sequence of the 2Fe-2S Spinacia oleracea ferredoxin was obtained from the NCBI database (accession number O04683). Panel A, Phylogenetic analysis of ferredoxins. The tree was constructed by the neighbor-joining method with the MEGA4 program [45]. Bootstrap values are shown at the nodes. Bar, evolutionary distance of 0.2. Panel B, Sequence alignment of ferredoxins from Methanosarcina species. Motifs predicted to ligate two 4Fe-4S clusters are highlighted. The alignment was performed with ClustalX2 [46]. [file 1471-2180-11-165-S2.TIFF]

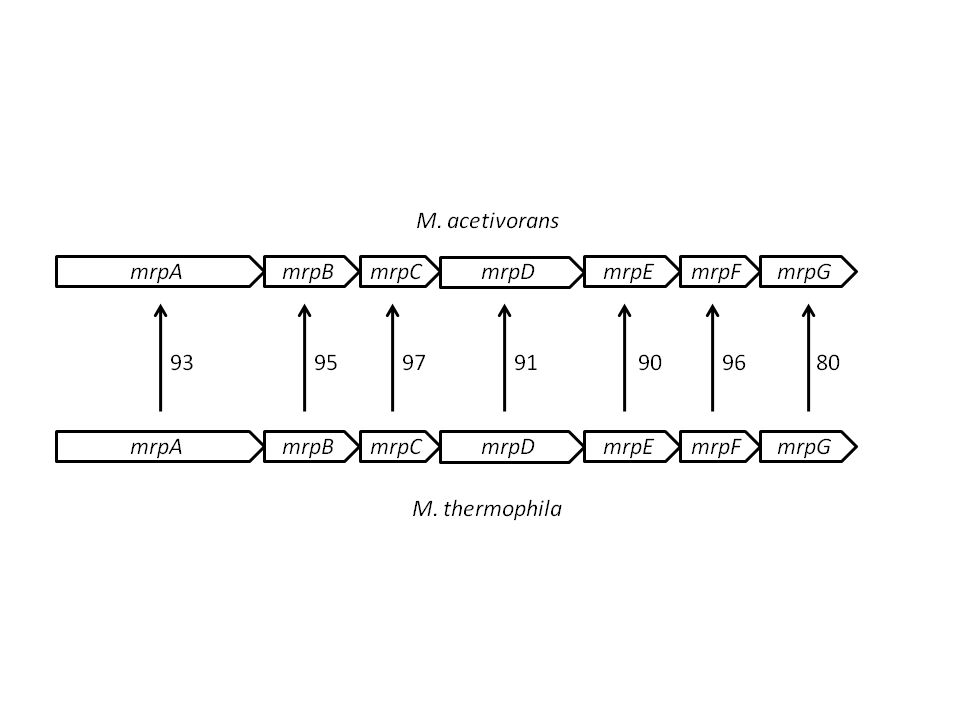

Supplement: Additional file 4 — Figure S4. Alignment of mrp gene clusters between Methanosarcina thermophila and Methanosarcina acetivorans. Numbers next to the arrows indicate deduced sequence identity. [file 1471-2180-11-165-S4.TIFF]
