# Supplementary material for: Electron transport in acetate-grown Methanosarcina acetivorans
Source: BMC Microbiol. 2011 Jul 24;11:165. doi: 10.1186/1471-2180-11-165 (PMC3160891; doi:10.1186/1471-2180-11-165)
Supplement: Additional file 3 — Figure S3. Comparison of rnf genes between Methanosarcina thermophila and Methanosarcina acetivorans. Panel A. Organization of rnf genes in Methanosarcina thermophila versus Methanosarcina acetivorans. Numbers next to the arrows indicate deduced sequence identity. Panel B. Alignment of the deduced sequences of rnf genes between Methanosarcina thermophila (Mt) and Methanosarcina acetivorans (Ma). Highlighted are: conserved heme binding sites (CXXCH and CXXXCH) in Cyt c, the flavin binding motif (SGAT) in RnfG, and cysteine motifs binding iron-sulfur clusters in RnfC and RnfB. [file 1471-2180-11-165-S3.PDF]

Figure S3

A

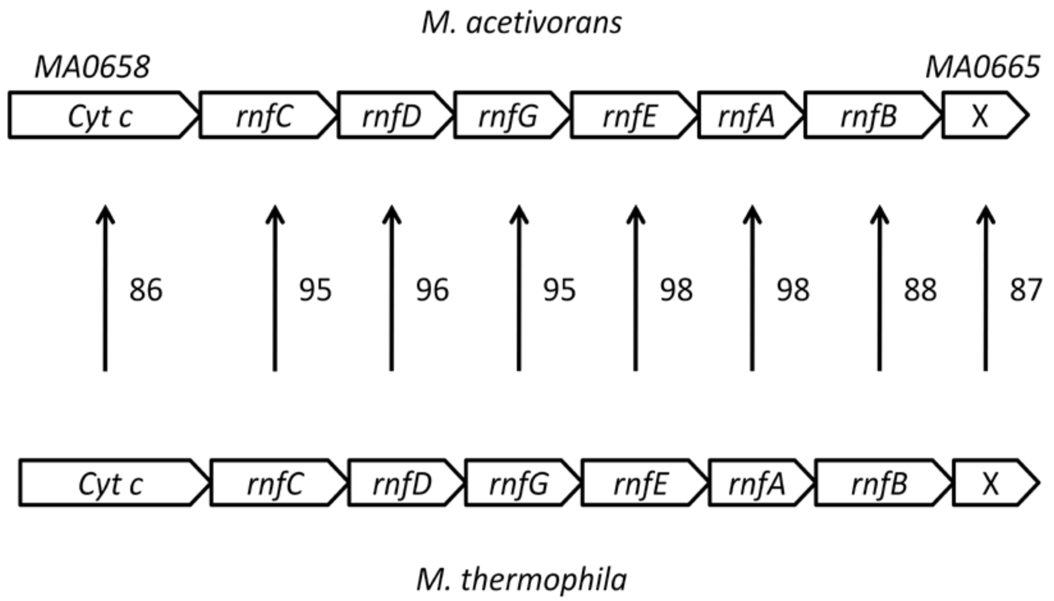

B

```
Mt Cyt c        ---MNRLNLLVSGVAVLLLMAAGAYSSLGYAGNDQIASHYMTKGEWSDTVCGGCHFDVYE
Ma Cyt c        MVIMNRLNLLVSGVAVLLLLAAGAYSSLGYSGNDAIASHYMTKGEWSDSVCGGCHFGVYE
                   ****************:**********:*** *************:*******.***

Mt Cyt c        NVNDSYHVQVDMNRWSPLTNFDLETSGEEEWVKEFGKYHPGGGPLAKYGIGIDCMMCHEQ
Ma Cyt c        NVNNSYHVQVNMSRWSPLTNFDLETSGEEEWVKKFGMYHPGGGPLAKYGIDIDCMMCHEK
                ***:******:*.********************:** *************.********:

Mt Cyt c        YGLYDSEARAEKIEEGDYEDANSAAMVNSSFAARTNPIQFFVYNANLLTPYPLLIVFHDN
Ma Cyt c        YGLYDFDARAEAIANGDFANANSLAVANFSATAQSDPLHLFVYTANVLTPYPLLIVFHDA
                ***** :**** * :**: :*** *:.* * :*:::*:::***.**:************

Mt Cyt c        VNGAPQESSCAEKCHITDVEPRAVSWGDEESYAESDVHAAKGVECTECHHTEGFIITSDH
Ma Cyt c        VNGAP--ISCAQRCHRIDVETSAVMWADEEDFEESDAHAANGVECTECHHTEAFIITSDH
                *****   ***::**  ***. ** *.***.: ***.***:***********.*******

Mt Cyt c        QIGRGNTSDSPDLPESHYDGTMRSCDDAECHAGISHGPFADSHMEFLACEACHIPKLPGG
Ma Cyt c        QIGRGNTSGTPDLPDSHYDDTMRSCDDAECHAGISHGPFADSHMEFLACEACHTPELPGG
```

```
                ********.:****:****.********************************* *:****

Mt Cyt c        DLPGGKPLESFSWQNGEREDVYREADFQPALAWYNGTFGDVLPSVDTRNDTDVMLTPFNN

Ma Cyt c        DLPGGNVLESFSWQNGEREDVYRDSDFQPALAWYNGNFGDVLPSVDTRNDTDVKVTPFNN

                *****: ****************::***********.**************** :*****

Mt Cyt c        ITGTWWDAGTDPEVLANPNTSISTGDPIPVQYVKAADANGDGEVTVEEMQAYDADGDGEA

Ma Cyt c        ITGTWWDAGTDPEVLANPNTSISTGDPIPVQYVKAADANGDGEVTVEEMQAYDADGDGEA

                ************************************************************

Mt Cyt c        DYPNAVLRTVELYYQVVHSIVSSDIGLADPYTCKDCHGNEAVIDWAALGYEQDPGGESSA

Ma Cyt c        DYPNAVLRTVELYYQVAHSIVSSDIGLADPYTCKDCHGNEAVIDWAALGYEQDPGGESSA

                ****************.*******************************************

Mt Cyt c        VKSIAVTYDRPKPVEVETEPAX

MA cyt c        VKSIAVTYDKPRPVEVETEPAL

                *********:*:*********


Mt RnfC        ------------LSDVIKIDKLPEKAIIPMRQHDGIACAPLVKKGDEVIVGQKLGECEGS

Ma RnfC        MKRSLHSKEVANLSDVIKIDKLPEKAIIPMRQHDGIACAPLVKKGAEVIVGQKLGECEGS

                            ********************************* **************

Mt RnfC        DLAYVHSPFCGTVNSIGLMPNPSGKRILSVVLTPSECEQTVDFIPEKNVPPSRLIEIIKE

Ma RnfC        DLAYVHSPFCGTVNSIELMPNPSGKRILSVVLTPSECAQTVDFVPEKDAPPSRLIEIIKE

                **************** ******************** *****:***:.***********

Mt RnfC        AGIVEYYEKPTYLALKPGKRIDTLLMNATFPLITHAYLSSLDKVLEGFKLMLEASGIPRG

Ma RnfC        AGIVEYYEKPTYLALKPGKRIDTLLMNATFPLITHAYLSSLDKVLEGFKLMLEASGISRG

                *********************************************************.**

Mt RnfC        VIVLRADDKESIKAFKNAKVDGKPLTVAPIVGMRHADYYLEDVEDQIIVVAAGNITYTPT

Ma RnfC        VIVLRADDKESIKAFKNAKVDGKPLTVAPIVGMRHADYYLEDVEDQIIVVAAGKITYTPT

                *****************************************************:******

Mt RnfC        MMNLLSANVMGRKLLLGHELPDVHVVVCGVKSAKAVYDAINEGKPYLESAVTVTGAVNNP

Ma RnfC        MMNLLSANVMGRKLPLGYEPPDVHVVVCGVKSAKAVYDAINEGKPYLESAVTVTGAVNNP

                ************** **:* ****************************************

Mt RnfC        KTVIVKFGTPIKDVIDACGGYKGEPGKVIINGSMGGVAVYTDEVPVVKNTVGIVVQTEAE

Ma RnfC        KTVIVKFGTPIKDVIEACGGYKGEPGKVIVNGSMGGVAVYTDEAPVVKNTVGIVVQTEAE

                ***************:*************:*************.****************
```

```
Mt RnfC      VLRDEATVCIHCARCVDVCPMNLLPGRIAAMADQGMFDRCKDYFALNCIECGECAVVCPA
Ma RnfC      VLRDEATVCIHCARCVDVCPMNLLPGRIAAMADMGMFDRCREYFALNCIECGECAVVCPA
             ********************************* ******::******************

Mt RnfC      KKHLVQLIRYSKLQIMNQKNETVEATX
Ma RnfC      KRHLVQLIRYSKLQIMNQKNETVEATE
             *:************************


Mt RnfD      MTSFTVSPPPHRKKKIFIKNLIWSRIFALLPISAAAVYFFGFAALGNLIASILGAVGIEF
Ma RnfD      MTSFTVSPPPHIKKKIFIKNLIWSRIVALLPISAAAVYFFGFAALGNIIASILGAVGIEF
             *********** **************.********************:************

Mt RnfD      VIQKAFNKKLTILDGNAIYLGLLLALISPPTLPAWMIFIGGAFAVGVGKHAFGGIGSYIF
Ma RnfD      VIQKAFNKKLTIMDGNAIYLGLLLALICPPTLPAWMIFIGGAFAVGVGKHAFGGIGSYTF
             ************:**************.****************************** *

Mt RnfD      HPSLAAWVFLSLAWAQDMLPGTIPILSSFSDLILENGAGFLTDVSPILVLLAGVILILVK
Ma RnfD      HPSLAAWVFLSLAWAQDMLPGTIPILSSFSDLILENGAGFLTDVSPILVLLAGVILILVK
             ************************************************************

Mt RnfD      YIEWRIPLSYLLTTVILALALGDPLAYVVSGTFLLGVFFLATETVTSPVTRNGRIVYGIL
Ma RnfD      YIEWRIPLSYLLTTVILALVLGDPLAYVVSGTFLLGVFFIATETVTSPVTQNGRIVYGIL
             *******************.*******************:**********:*********

Mt RnfD      CGFLTVVYGYFSGNYVWGTLYALLLSNAVAPFIELKTLPKPMGGVADE
Ma RnfD      CGFLTVIYGYFSGNYVWGTLYALLLSNAVAPFIELKTLPKPMGGVANE
             ******:***************************************:*


Mt RnfG      MSDSKEITKVIVTIVVISAVAAALLALTYTPTQAKLELLQAEQQKEAMKAILPQASDFEP
Ma RnfG      MSDSKEITKVIVTMVVISAVAAALLALTYTPTQAQLKLLQAEQQKEAMKEILPQAADFEP
             *************:********************:*:************ *****:****

Mt RnfG      VTGSEVDDDGNPVVLYYRGVDSSGNVVGYVVERNQVGAQGMIQLLAGISSDFGTITGFQV
Ma RnfG      VTGSEVDDDGNPVVLYYKGVDSSGNVVGYVVERNQVGAQGMIQLLAGISSDFSTITGFQV
             *****************:**********************************.*******

Mt RnfG      MKHSETPGLGALITTPEFQGQFVDLPVADTSLTKNGGQVDAISGATISSQAVVDALHSAV
Ma RnfG      MKHSETPGLGALITTPEFQGQFVDLPVADTSLTKNGGQVDAISGATISSQAVVDALHSAV
             ************************************************************
```

```
Mt RnfG         DYVSAQEG
Ma RnfG         DYVSAQEG
                ********


Mt RnfE         MYPHRRADMNPISEFIRGITKDNPTFGLVLGLCPTLAVTTSVENGIGMAMGTLFVLVGSN
Ma RnfE         MYPHRRADMNPISEFIRGITKDNPTFGLVLGLCPTLAVTTSVENGIGMAMGTLFVLVGSN
                ************************************************************
Mt RnfE         MMVSAIRKGIPGTVRLPIEIIVIATFVTIVDMVMEAFTPDLYASLGVFIPLIVVNCIVIG
Ma RnfE         MMVSAIRKGIPGTVRLPVEIIVIATFVTIVDMVMEAFTPDLYTSLGVFIPLIVVNCIVIG
                *****************:************************:*****************
Mt RnfE         RAEAYALKNGVFYSIIDALGEGTGFLLVLILIGGIRELLGTGIIDPFGMTLINLSGIITP
Ma RnfE         RAEAYALKNGVFYSIIDALGEGTGFLLVLILIGGIRELLGTGIIDPFGMTLINLSGVINP
                ********************************************************:*.*
Mt RnfE         AMFMTMSPGAFLTIAVLMTIVNYRRQQKAAKGG
Ma RnfE         AMFMTMSPGAFLTIAVLMTIVNYRRQQKAAKGG
                *********************************


Mt RnfA         VVKMAESLFTIFLEGVFIKNFLLIQFLGLCSFVGVTKDLKSASGMSGAVVFVMAMAATVS
Ma RnfA         MVKMAESLFTIFLEGVFIKNFLLIQFLGLCSFVGVTKDLKSASGMSGAVVFVMAMAATVS
                :***********************************************************
Mt RnfA         FALYNFILVPLKLEFLRTIAFIVVIAALVQLVEFIVRKHVPALYRSLGIYLPLITTNCAV
Ma RnfA         FALYNFILVPLKLEFLRTIAFIVVIAALVQLVEFIVRKHVPALYRSLGIYLPLITTNCAV
                ************************************************************
Mt RnfA         LGAVLLNVMNDYNFAQSVVFGVAAGLGYTVAMLMMAAIRERSDLVEVPKSVGRGVTYAFF
Ma RnfA         LGAVLLNVMNDYDLAQSVVFGVAAGLGYTVAMLMMAAIRERSDLVEVPKSVGRGVTYAFF
                ************::**********************************************
Mt RnfA         IATIMSMSFVNFFGVIPLE
Ma RnfA         IATIMSMSFVNFFGVIPLE
                *******************
```

```
Mt RnfB         LSDVLINSIAVLAGLGFAVGVMLVIASKVFKIDSNPLIDDVASLLPGANCGGCGFAGCAA
Ma RnfB         MSSVLINSIAVLAGLGFAVGVMLVIASKVFKIDSNPLIDDVASLLPGANCGGCGFAGCAA
                :*.*********************************************************
Mt RnfB         CAEAIVEQGAPINSCPVGGFEVAKQIGALLGQEVTESEKAFPFVRCQGGQAHCTTLYDYH
Ma RnfB         CAEAIVEQGAPVNSCPVGGFEVAKQIGALLGQEVTESEKEFPFVRCQGGNQHCTTLYDYH
                ***********:*************************** *********: *********
Mt RnfB         GVEGCKAALMLCDSKKGCTYGCIGLGTCVRACQFDALSMGEDGFPVVNKNLCTSCGNCIA
Ma RnfB         GVENCKVALMLCDSRKGCTYGCLGLGTCVQACQFGALSMGEDGFPVVNKALCTSCGNCIA
                ***.**.*******:*******:******:****.************** **********
Mt RnfB         ACPNGILTFARDSEKVHVLCRSHDKGKDVKAVCEVGCIGCKKCEKECPTGAIKVTNFLAE
Ma RnfB         ACPNGVLTFARDSEKVHVLCRSHDKGKDVKAVCEVGCIGCKKCEKECPAGAIRVTEFLAE
                *****:******************************************:***:**:****
Mt RnfB         IDQEKCTACGACVAICPQKSIELR
Ma RnfB         IDQEKCTACGACVAICPQKAIELR
                *******************:****
```

```
Mt RnfX         MPYKTFLGLPENIVAALCYPVGWLSGLFFLLLERKNKFVRFHAMQSVLLFMPYVLFLFLV
Ma RnfX         MSYNTSLGLSENIVAALCYPVGWLSGLFFLLLERKNKFVRFHAMQSVLLFMPIALFIFLV
                *.*:* ***.****************************************** .**:***
Mt RnfX         AWIPTIGWAIADSVGMPGMLLIVIPMYMAFRGSKFKIPIIGKIAYNFAYGE
Ma RnfX         AWIPTIGWFIADGAGMTAMLLILIPMYMAFRGSKFKIPIIGNIAYNFAYGE
                ******** ***..**..****:******************:*********
```
